# Journal of Clinical Monitoring and Computing 2019 end of year summary: monitoring tissue oxygenation and perfusion and its autoregulation

**DOI:** 10.1007/s10877-020-00504-z

**Published:** 2020-04-10

**Authors:** M. M. Sahinovic, J. J. Vos, T. W. L. Scheeren

**Affiliations:** Department of Anesthesiology, University of Groningen, University Medical Center Groningen, Hanzeplein 1, PO Box 30001, 9700RB Groningen, Netherlands

**Keywords:** Monitoring, Cerebral tissue oxygenation, NIRS, Cerebral autoregulation, Cerebral blood flow, Somatic tissue oxygenation

## Abstract

Tissue perfusion monitoring is increasingly being employed clinically in a non-invasive fashion. In this end-of-year summary of the Journal of Clinical Monitoring and Computing, we take a closer look at the papers published recently on this subject in the journal. Most of these papers focus on monitoring cerebral perfusion (and associated hemodynamics), using either transcranial doppler measurements or near-infrared spectroscopy. Given the importance of cerebral autoregulation in the analyses performed in most of the studies discussed here, this end-of-year summary also includes a short description of cerebral hemodynamic physiology and its autoregulation. Finally, we review articles on somatic tissue oxygenation and its possible association with outcome.

## Introduction

In the last decade, perioperative tissue perfusion monitoring has become an emerging monitoring modality mainly because it enables evaluating the coupling of macro-hemodynamic variables with regional or local hemodynamics at the tissue of interest. Tissue perfusion monitoring is, due to its (increasing) ease of use and reliability, gradually used perioperatively, predominantly for monitoring of cerebral perfusion. Additionally, tissue perfusion monitoring may also be used for monitoring somatic tissues, e.g. muscular, renal or splanchnic perfusion.

In this end-of-year review, we discuss the latest studies concerning this subject that were published last year in the Journal of Clinical Monitoring and Computing. As most of these studies focus on cerebral perfusion monitoring, we will first discuss relevant topics on cerebral perfusion, including cerebral autoregulation (CA), and how these can be monitored. Please see Table 1 for a list of abbreviations used in this review.

**Table 1:** List of abbreviations

ABP	Arterial blood pressure
AUC	Area under the curve
BCP	Beach-chair position
Ca	Cerebral arterial compliance
CA	Cerebral autoregulation
CBF	Cerebral blood flow
CBFV	Cerebral blood flow velocity
ccCBF_MRpuls_	Calculated change in blood flow estimate
CCT	Cerebral circulation time
CFF	Continuous flow forward
CPP	Cerebral perfusion pressure
CrCP	Critical closing pressure
DCM	Diastolic closing margin
DoA	Depth-of-anesthesia
EEG	Electroencephalogram
etCO_2_	End-tidal concentrations of carbon dioxide
ICP	Intracranial pressure
MAP	Mean arterial pressure
MCA	Middle cerebral artery
MMSE	Mini mental state examination
Mxa	Mean flow index
NIRS	Near-infrared spectroscopy
OR	Odds ratio
PDA	Patent ductus arteriosus
PFF	Pulsatile flow forward
POCD	Postoperative cognitive dysfunction
PONV	Postoperative nausea and vomiting
ROC	Receiver operating characteristic
rSO_2_	Regional tissue oxygen saturation
SAH	Subarachnoid hemorrhage
ScO_2_	Cerebral oxygen saturation
StO_2_	Tissue oxygen saturation
TCD	Transcranial doppler
ΔCaBV	Changes in cerebral blood volume
ΔV	Change in volume
τ	Time constant of the cerebral arterial bed

Monitoring of cerebral perfusion and CA can provide vital insight into physiology and pathophysiology of intracranial processes and diseases and can thereby help guide therapies and improve outcomes, especially within the context of perioperative medicine [1]. CA is a physiological mechanism that maintains stable cerebral perfusion and brain tissue oxygenation in face of changing cerebral perfusion pressure (CPP). In normotensive individuals, CA keeps cerebral blood flow (CBF) relatively constant in the range of a mean arterial pressure (MAP) of approximately 60–150 mmHg [2]. Within these limits, CBF is kept constant by regulatory capabilities of cerebral vessels while outside these limits, flow becomes pressure dependent and cerebral hypo- or hyperperfusion can occur [3]. CA can be divided into a static and a dynamic component. Static autoregulation describes the extent to which the vascular bed can constrict or dilate while dynamic autoregulation also includes the rate at which these changes occur [4]. CA is a fast accommodating process. Several monitoring modalities which are capable of tracking these alterations continuously have been developed.

**Fig. 1:**
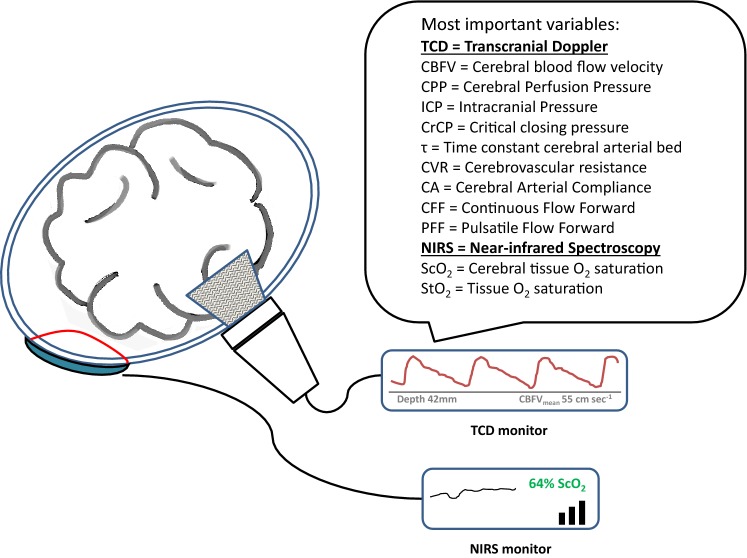
Graphical repesentation of two commonly used cerebral hemodynamic monitoring modalities: Transcranial Doppler (TCD) and Near-Infrared Spectroscopy (NIRS)

Noninvasive near-infrared spectroscopy (NIRS; Fig. 1) allows bedside monitoring of (cerebral) tissue oxygenation by measuring the ratio between oxy- and deoxyhemoglobin saturation in the small vessels (< 150 µm) at the site of measurement. Hence, the obtained tissue oxygen saturation (StO_2_) resembles the microvascular oxygenation status of the investigated tissue(s). Technical details on NIRS monitoring were discussed previously [5].

Noninvasive transcranial Doppler (TCD; Fig. 1) measures the cerebral blood flow velocity (CBFV) in the middle cerebral artery (MCA). Using TCD derived data and mathematical modeling, several TCD based indices have been developed that can be used to classify and quantify the status of the CA [6].

The critical closing pressure (CrCP) is the value of the arterial blood pressure at which CBF approaches zero. It has been predominantly studied and measured in animal models and only rarely in humans. There are several models available to estimate CrCP in vivo. For an extensive discussion of CrCP, please see [7].

The time constant of the cerebral arterial bed (τ) is a hemodynamic measure of CBF impairment. Conceptually, τ can be thought of as the time needed to stabilize the cerebral blood volume after a sudden change in arterial blood pressure (ABP) during one cardiac cycle; it is, to some extent, analogous to cerebral arterial compliance (Ca). However, contrary to the latter, the calculation of τ is not dependent on the cross-section of the insonated intracranial vessel, and thus, it can be directly compared between patients. Its effectiveness has been investigated in patients with carotid stenosis and in patients with subarachnoid hemorrhage (SAH). τ was shorter in patients with unilateral (t = 0.18 ± 0.04 s) and bilateral (t = 0.16 ± 0.03 s) internal carotid stenosis compared to the controls, and it correlated with the degree of stenosis [8].

In patients with SAH, τ shortening secondary to vasospasm could be observed before formal TCD signs of vasospasm occurred, and thus could potentially be used as early warning sign of an impending vasospasm, thereby allowing more time to initiate treatment [9]. For an extensive discussion of τ, please read [10].

## Assessment of cerebral hemodynamic variables using pulsatile versus non-pulsatile cerebral blood outflow models

In the article published by Uryga and colleagues in the Februari 2019 issue, the authors aimed to assess two cerebral hemodynamic indices, namely the CrCP and τ in healthy volunteers, during alterations of end-tidal carbon dioxide concentrations (etCO_2_) [11].

These hemodynamic indices were estimated using two different models which were developed to evaluate the changes in cerebral blood volume (ΔCaBV) based on the arterial cerebral blood flow velocity measured by TCD. These cerebral blood outflow models were the continuous flow forward model (CFF) and the pulsatile flow forward model (PFF). CFF assumes that a continuous flow can approximate CBF distal to the big cerebral arteries while the PFF model presumes that a pulsatile flow can best approximate this flow. The indices were estimated by the mathematical transformation of the pulse waveforms of the ABP and CBFVa. The authors conclude that the PFF model better reflects changes in CrCP and τ induced by CO_2_ alterations.

In a related article published by the same authors in the August 2019 issue, they compared τ calculations from the middle and posterior cerebral arteries in 32 healthy subjects [12]. They found that the PFF model estimated shorter τ compared to the CFF model. Furthermore, τ obtained using the PFF model was significantly longer in the middle cerebral artery than in the posterior cerebral artery. Their results suggest that τ calculated by the PFF model better reflects the cerebral vascular differences in the different brain lobes compared to τ derived from the CFF model.

## Intracranial pressure curves correlate with cerebral blood flow

The current treatment guidelines for patients with severe traumatic brain injury recommend monitoring and maintenance of CPP between 60 and 70 mmHg to optimize CBF and improve the patients` outcome [13]. CPP is defined as the difference between MAP and intracranial pressure (ICP). However, CPP is merely a reflection of the potential driving pressure for cerebral perfusion and does not necessarily reflect the actual blood flow; thus, a more direct method of estimating CBF is needed.

In the February 2019 issue, Unnerbäck et al. [14] investigated a method which could potentially achieve this by analyzing the intracranial pressure (ICP) curves. By using the cerebral elastance coefficient (i.e. the relation between intra-cranial pressure and volume) they converted the ICP curve into a “change in intracerebral volume” (ΔV) curve. Further, by integrating this curve over one cardiac cycle, they have calculated the “area under the curve” for ΔV (AUC_ΔV_). Subsequently, they compared this “calculated change in blood flow estimate” to the pulsatile part of CBF (ccCBF_MRpuls_), measured during one cardiac cycle, by “phase contrast” magnetic resonance imaging. They found a correlation between the AUC_ICP_ and ccCBF_MRpuls_ (R^2^ = 0.440; p = 0.013). The AUC_ΔV_ correlated more appropriately with the ccCBFMRpuls (R^2^ = 0.688; p < 0.001). They concluded that the pulsatile part of the ICP curve, and especially when integrated into a volume curve, correlated with the pulsatile component of the CBF as measured by magnetic resonance imaging.

## Postural change(s) and cerebral perfusion

In the August 2019 issue, Cardim et al. [15] investigated cerebral hemodynamics in patients (n = 23) undergoing shoulder surgery in the beach-chair position (BCP). In BCP, the cranial part of the operating table is tilted to an angle between 45–90° (also called sitting position), so that the shoulder is optimally exposed to the surgical team. As expressed by the authors, gravity may exaggerate the effects of hypotension (induced by BCP itself) on CPP in BCP, since unlike in the supine position, the head is above the level of the heart. The authors assessed the relationship between ABP—continuously measured using a finger cuff, calibrated at the auditory meatus level—and CBFV of the MCA, assessed using TCD. Measurements were done both in supine position and in BCP, after induction of general anesthesia. The authors not only studied CBFV itself but also calculated surrogates of intracranial pressure (“noninvasive” ICP) and CA. For ICP, actual ABP values were used as input to a so called ‘black box model’ together with reference CBFV values (from patients with traumatic brain injury). The model, described extensively elsewhere [16], produced a continuous waveform of ICP—used as surrogate of true (invasively determined) ICP. Hence, CPP could be calculated as well, which also allowed calculation of the diastolic closing margin (DCM), which equals ABP-CrCP, and identifies the range of ABP values that would endanger cerebral perfusion once DCM would get closer to zero. For evaluating CA, another model was used by the authors, and Mxa—the “mean flow index”—was calculated, representing the dependency of CBFV (–derived variables) on fluctuations in ABP. After induction of general anesthesia, ABP was decreased, both in supine position and BCP. Although it was not significant, the ABP tended to be lower once patients were positioned in BCP (62 mmHg vs. 49 mmHg, p = 0.21). Strikingly, both in supine position and in BCP, CA was suggested dysfunctional according to the Mxa value. Also, after positioning in BCP, DCM decreased significantly, but did not reach 0 mmHg. Additionally, ICP tended to decrease after BCP institution. The findings from this study may tell us the following: after induction of general anesthesia, hypotension developed, and CA was dysfunctional, which may be secondary to either the administration of anesthetics, or to ABP values being out of individual range for maintaining CA. Secondarily, as based on these TCD-derived calculations, BCP further endangered cerebral perfusion, as DCM decreased substantially – in some patients to values of about 5 mmHg—and was correlated with ABP values. Hence, these results may potentially allow an individualized hemodynamic optimization for maintaining cerebral perfusion in BCP, if only these measurements were more easily obtained. For now, the results from this study emphasize that prevention of intraoperative hypotension in this specific surgical procedure should not only aim at the prevention of myocardial and renal injury, but also at the prevention of potentially catastrophic cerebral events [17], and requires careful and continuous ABP monitoring and management in patients placed in BCP, in order to maintain CPP.

## Cerebral circulation time

Sevoflurane was traditionally frequently used for maintenance of anesthesia during neurosurgery while nowadays, due to its favorable pharmacology, the use of propofol is on the rise [18]. In the study published in December 2019 edition of the JCMC, Ishibashi and colleagues investigated the influence of the propofol versus sevoflurane on cerebral circulation time (CCT) [19]. They performed this study in patients undergoing elective coiling of an unruptured cerebral aneurysm, who were randomized in two groups: The first group received propofol for the first part of the procedure (until after the first angiogram), after which the hypnotic was switched to sevoflurane for the second part of the procedure (and the second angiogram). The second group received the same drugs but in reverse sequence. By standardizing the injection site, infused volume, and injection speed of contrast medium and by measuring the time to peak density of contrast medium at different regions of interest in the arterial and venous cerebral vessels, they determined the CCT through different parts of cerebral vessels, across individuals. They performed three CCT measurements; the first one before the induction of anesthesia (baseline) and the second/third measurements during anesthesia which was maintained with differing drugs. They found that CCT (median [interquartile range]) was 10.9 (9.65–11.98) s under propofol-based anesthesia compared with 8.78 (8.32–9.45) s under sevoflurane-based anesthesia (P < 0.001). Circulation times for the internal carotid, middle cerebral artery, and microvessel segments were also longer under propofol-based anesthesia than under sevoflurane-based anesthesia. Their findings suggest that, at comparable BIS-guided anesthetic depths, propofol proportionately decreases CBF more than sevoflurane.

## Predicting delayed cerebral ischemia after subarachnoid hemorrhage

Current tools used for predicting the occurrence of delayed cerebral ischemia after SAH rely on imaging made at hospital admission. They are simple to employ; however, their predictive ability is rather low. There is thus a need for better prediction models.

In the article published by Park and colleagues in the February 2019 issue [20], the authors set out to develop and validate a new prediction model, using a temporal unsupervised feature engineering approach. For model development, they used data collected retrospectively from 488 patients admitted to their unit/hospital with a SAH. Their derivation dataset, which was 80% of all available data, consisted of demographic information, established SAH grading scales, and features from physiological time series (e.g., blood pressure, heart rate), which were extracted using random kernels. They trained different classifiers on this dataset to predict a dichotomous outcome. Subsequently, they applied their model to the validation dataset (remaining 20% of the data). Model prediction accuracy based on grading scales alone achieved an AUC of 0.58, while a model based on combined baseline data, grading scales, and physiologic data produced the best classification performance with an AUC of 0.77. This publication shows that, using agnostic and inexpensive machine learning, predictive models can be developed that have the potential to improve patient care.

## Near-infrared spectroscopy (NIRS) for monitoring cerebral and peripheral tissue oxygenation

Finally, another cerebral monitoring tool—NIRS—was used in the study by Karademir et al. [21] in the October 2019 issue. In their study, cerebral oxygen saturation (ScO_2_) was monitored by NIRS in normotensive (n = 25) and pre-eclamptic (n = 24) pregnant women during spinal anesthesia for caesarean section. In normal pregnancy, TCD has shown decreased (systolic) flow velocity, as well as decreased resistance index, with an up to 50% increase in CPP [22]. Due to intact CA, CBF changed to a much lesser extent. In pre-eclampsia, however, especially in severe cases, CA may fail, leading to potentially catastrophic cerebral events. It was the authors` aim to study the effects of spinal anesthesia on ScO_2_ in these two patient categories. In both normotensive and pre-eclamptic patients, ScO_2_ baseline values were similar, and decreased temporarily to some extent after the application of spinal anesthesia. A subgroup analysis revealed that in patients with more severe pre-eclampsia (i.e., those patients with an increased likelihood of failing CA), baseline ScO_2_ values were somewhat higher (68% vs. 62%, not tested for significance). The clinical significance of the authors’ findings in these awake pregnant women is undetermined; future studies that incorporate NIRS and TCD-derived cerebral hemodynamics may further elucidate the exact influence of anesthetic management, especially in those patients at increased risk of complications (i.e., those with severe pre-eclampsia).

A further interesting case report on the use of *somatic* NIRS measurements was reported by Saito et al. in the June issue of the journal [23]. They presented a case of a 14-day-old infant with a patent ductus arteriosus (PDA) which was going to be clipped. During surgery for PDA ligation, the authors measured somatic regional tissue oxygen saturation (rSO_2_) by NIRS on the patient’s back. After clipping, the rSO_2_ values decreased from 65 to 55% instead of the expected increase. Shortly after surgery, echocardiography revealed that the PDA was still open. Subsequent computed tomography revealed that accidental clipping of the upper left pulmonary artery had taken place, and the patient went for urgent re-thoracotomy. After the clip was released from the left pulmonary artery and the „true “ PDA was clipped, rSO_2_ values increased from 67 to 83%. The authors discussed that retrospectively, the unexpected change in rSO_2_ values might have been a key to the early diagnosis of accidental clipping of the wrong artery in this case, and they conclude that the somatic rSO_2_ should be monitored routinely in PDA patients.

In the December issue, the same group of authors [24] published a retrospective observational study in which they studied the predictability of cerebral and renal rSO_2_ values obtained at the beginning of surgery for outcomes in 59 pediatric patients having cardiac surgery under cardiopulmonary bypass. Outcomes included 30-day mortality, need for renal replacement therapy or extracorporeal membrane oxygenation, and duration of mechanical ventilation and ICU stay. Overall, the rSO_2_ values (both cerebral and renal) were significantly lower in the patients with poor outcomes compared to those without poor outcomes. Receiver operating characteristic (ROC) analysis revealed that both cerebral and renal rSO_2_ were good predictors of each outcome, with cerebral values being superior to renal ones for some outcomes. For instance, a cut-off value of 51% cerebral rSO_2_ predicted 30-day mortality with good sensitivity and specificity. Of note, renal rSO_2_ values were associated with outcomes in both cyanotic (n = 31) and non-cyanotic (n = 28) patients, whereas cerebral rSO_2_ values were associated with outcomes only in cyanotic patients. The authors speculated that impaired CA in the cyanotic patients was responsible for this difference. The authors conclude that cerebral and renal rSO_2_ values obtained at the beginning of surgery might be useful for predicting the outcome of patients having congenital heart surgery.

In the December issue of the journal, Momeni et al. [25] published a large prospective observational study of 1.616 patients having cardiac interventions with or without general anesthesia, the majority having cardiac surgery. The aim of the study was to investigate the impact of electroencephalogram (EEG) suppression as assessed with a depth-of-anesthesia (DoA) monitor, and decreased cerebral rSO_2_ on the incidence of postoperative delirium and cognitive dysfunction (POCD). The authors used an algorithm to optimize cerebral oxygenation and kept the processed EEG index of the DoA monitor between 40 and 60. Postoperative neurological testing included a validated chart method for delirium and the Mini Mental State Examination (MMSE) for POCD. Furthermore, patients were followed up by telephone interview at 3 and 6 months postoperatively. Both delirium and POCD occurred in 20% of the patients each. Patients that experienced delirium had significantly more and longer periods of EEG suppression and also lower rSO_2_ values at the end of surgery. Similarly, patients presenting POCD had experienced more EEG suppression and showed more episodes of rSO_2_ decline and also lower rSO_2_ values at the end of surgery. Having experienced high magnitudes of EEG suppression was significantly associated with the likelihood of developing delirium, so was advanced age and previous alcohol abuse. For POCD, independent risk factors included low rSO_2_ values at the end of surgery, delirium, and advanced age. It has to be mentioned that due to the algorithm used to optimize cerebral oxygenation, episodes of cerebral desaturation (defined as a decrease in rSO_2_ of > 25%) were rather rare. Regarding EEG suppression, which might have been caused by either too deep anesthesia or cerebral hypoperfusion, a causal relation to the observed neurological alteration could not be proven by this study. Nevertheless, the study is an important contribution to the discussion whether a combined DoA and NIRS monitoring should be performed in patients having cardiac surgery. It should be noted also that due to recent recommendations the term “perioperative neurocognitive disorders” should be used fort he observed neurological disorders, which include POCD, delayed neurocognitive recovery and postoperative neurocognitive disorder [26].

While most studies reviewed here focus at more complex aspects of cerebral perfusion monitoring, the study by Li et al. [27] published in the August 2019 issue investigated a relatively “simple” issue of direct clinical relevance: the association between intraoperative muscular tissue oxygenation and the postoperative occurrence of postoperative nausea and vomiting (PONV) within 24 h after robotic (laparoscopic) hysterectomy. In short, the authors prospectively enrolled 106 patients, and measured oxygenation of the brachioradial muscle (located on the radial side of the forearm). First, the authors determined baseline StO_2_, while patients were still awake. This baseline StO_2_ was used to determine different threshold values under the respective baseline (5–20% under baseline), as well as absolute StO_2_ values—all determined in the intraoperative phase. Additionally, the authors calculated the AUC below the baseline. A total of 35 patients (33%) experienced any form of PONV within 24 h after surgery. The authors found an association between forearm StO_2_ and PONV occurrence: e.g. patients in whom StO_2_ decreased > 15% below individual baseline had an associated odds ratio (OR) of 2.8 (95% CI 1.05–7.43), with an increase in OR for an StO_2_ decrease < 20% below baseline to 13.8 (95% CI 2.83–67.38) for developing PONV. Similar values were found for AUC values and PONV, while in contrast, in patients in whom forearm StO_2_ was increased intraoperatively, the odds of experiencing PONV were reduced. The observed association between intraoperative forearm StO_2_ and PONV is sound, but does not necessarily imply causation. It is well known that the occurrence of PONV is related to multiple factors, including intraoperative hemodynamic alterations such as hypovolemia [28] and hypotension [29]. Hence, the authors` suggestion that forearm StO_2_ may be seen as a *surrogate* for gastro-intestinal tissue oxygenation, and may be used as a “target” for the prevention of PONV (assuming that suboptimal gastro-intestinal tissue oxygenation is the reason for PONV), requires further verification. To proof the actual correlation of forearm StO_2_ and gastro-intestinal tissue oxygenation would require measuring both variables simultaneously, which in turn would require the application of a (sterile) probe on the gut, preferably in a continuous fashion (e.g. during ongoing surgery). Clearly, this is not very feasible. Moreover, the delicate balance between anesthetic drugs (e.g., hypnotics, analgetics) and the resulting depth of anaesthesia, macro-hemodynamics (e.g., arterial blood pressure, fluid responsiveness), tissue oxygenation, and occurrence of PONV remains to be elucidated. As suggested by the authors, actually targeting forearm StO_2_ above a certain threshold may aid in discriminating whether this would reduce the incidence of PONV. Importantly though, this would require the standardization of the anesthetic technique incorporating StO_2_ targets, and should preferably be done in the context of goal-directed hemodynamic optimization, as has been done before [30].
